# Expression of xylanase XynB is synergistically controlled by two two-component systems in *Ruminiclostridium cellulolyticum*

**DOI:** 10.1128/aem.00062-25

**Published:** 2025-05-30

**Authors:** Wenhao Zhang, Zili Qiu, Qiuyun Zhao, Ziyi Liu, Xiaorong Zhang, Houhui Song, Chenggang Xu

**Affiliations:** 1 College of Veterinary Medicine, Zhejiang A&F University 722545, Hangzhou, Zhejiang, China; 2 Key Laboratory of Applied Technology on Green-Eco-Healthy Animal Husbandry of Zhejiang Province, Hangzhou, Zhejiang, China; 3 Zhejiang Provincial Engineering Research Center for Animal Health Diagnostics & Advanced Technology, Hangzhou, Zhejiang, China; 4 Zhejiang International Science and Technology Cooperation Base for Veterinary Medicine and Health Management, Hangzhou, Zhejiang, China; 5 Key Laboratory of Chemical Biology and Molecular Engineering of Ministry of Education, Institute of Biotechnology, Shanxi University 631296 , Taiyuan, Shanxi, China; 6 Institute of Applied Chemistry, Shanxi University 631309, Taiyuan, Shanxi, China; Washington University in St. Louis, St. Louis, Missouri, USA

**Keywords:** *Ruminiclostridium cellulolyticum*, GH11 xylanase XynB, two-component systems, synergistic regulation

## Abstract

Xylan, a major component of hemicellulose, is crucially targeted by xylanases for its breakdown. This study focuses on the free xylanase XynB from *Ruminiclostridium cellulolyticum* to elucidate its expression and regulatory mechanisms. We successfully achieved heterologous expression and purification of recombinant XynB, verifying its enzymatic activity specifically against xylan. The mutation of *xynB* confirmed its essential role in xylan degradation by *R. cellulolyticum*. We further explored the transcription of *xynB* under various carbon sources and uncovered its regulatory mechanisms mediated by two-component systems (TCSs). We found that *xynB* transcription is activated by the xylan-sensing TCS (XuaDRS) and repressed by the cellobiose-sensing TCS (CuaDRS). This research enriches our understanding of the regulatory mechanisms governing the activity and expression of free xylanases like XynB from *R. cellulolyticum*, offering potential targets for the genetic engineering and process optimization of cellulolysis.

**IMPORTANCE:**

*Ruminiclostridium cellulolyticum*, an anaerobic, mesophilic, and cellulolytic gram-positive bacterium, is a model organism for the microbial degradation of plant cell wall polysaccharides and a promising host for biofuel production from lignocelluloses. The degradation process of lignocellulosic materials is complex due to their intricate structure and interlocking complexity. XynB, a GH11 family xylanase, plays a significant role in the breakdown of xylan, a major constituent of hemicelluloses. Our study reveals the molecular mechanisms that link the specific adaptation of xylan utilization with the general stress response in the regulatory network of *R. cellulolyticum*, particularly by detailing the synergistic effects of two two-component systems on the transcriptional regulation of *xynB*. This knowledge is essential for harnessing the full potential of *R. cellulolyticum* in the production of biofuels from lignocellulosic biomass.

## INTRODUCTION

Lignocellulosic biomass is the most abundant renewable resource on earth that can be converted into fermentable sugars for the production of cellulosic bioproducts (1). Lignocellulose is mainly composed of cellulose, hemicellulose, and lignin (2, 3). Hemicellulose, which can account for up to 35% of the total dry mass of the plant cell wall, is a heterogeneous group of branched and linear polysaccharides (4). Xylan stands out as a major constituent of hemicelluloses, a polysaccharide characterized by a backbone made up of β-1,4-xylose units (5). This backbone is often decorated with various side chains such as arabinose, methylglucuronic acid, and acetate groups (6, 7).

The degradation of the xylan backbone is primarily facilitated by endo-β-1,4-xylanases, which cleave the internal glycosidic bonds within the xylan chain, converting long-chain xylans into a variety of oligosaccharides (8, 9). However, the complete deconstruction of xylan into its constituent monosaccharides requires a suite of accessory enzymes, such as α-L-arabinofuranosidase, α-glucuronidase, β-xylosidase, and xylan acetyl esterase, which act on the side chains (10). In the Carbohydrate-Active enZYmes (CAZy) database (www.cazy.org), based on the similarity of amino acid sequence and catalytic cleft structure, xylanases have been classified into 17 glycoside hydrolase (GH) families (11). Among these, the GH11 family exhibits several interesting features compared to other xylanases: small size (around 20 kDa), high substrate selectivity and high catalytic efficiency, variety of pH and temperature optimum, so better fiber penetration can be achieved (12). The advantageous properties of the GH11 family make these enzymes a suitable candidate for the bio-refining of lignocellulosic materials, biofuel production, pulp production, green catalysts, and other industrial fields (13–15).

*Ruminiclostridium cellulolyticum*, an anaerobic, mesophilic, and cellulolytic gram-positive bacterium, is a model organism for the microbial degradation of plant cell wall polysaccharides and a promising host for the production of biofuels from lignocelluloses (16–19). Like many other cellulolytic bacteria, *R. cellulolyticum* H10 produces cellulosomes, which are cellulase complexes consisting of up to dozens of subunits that functionally cooperate and are the fastest protein molecular machines in nature for degrading cellulose (20–22). It harbors a distinctive gene CCEL_RS09445 encoding GH11 family xylanase (named XynB), which is transcribed at very high levels when xylan and glucose are present. In this study, we successfully overexpressed XynB and confirmed its enzymatic activity *in vitro*. We further analyzed its transcription under different carbon sources and determined its regulatory mechanism mediated by two-component systems (TCSs). This study enriches the basic understanding of the regulatory mechanism governing the activity and expression of free xylanases like XynB derived from *R. cellulolyticum* H10, which can be exploited for process and genetic engineering of cellulolysis.

## RESULTS

### *R. cellulolyticum* harbors two GH11 xylanases

**Fig 1:**
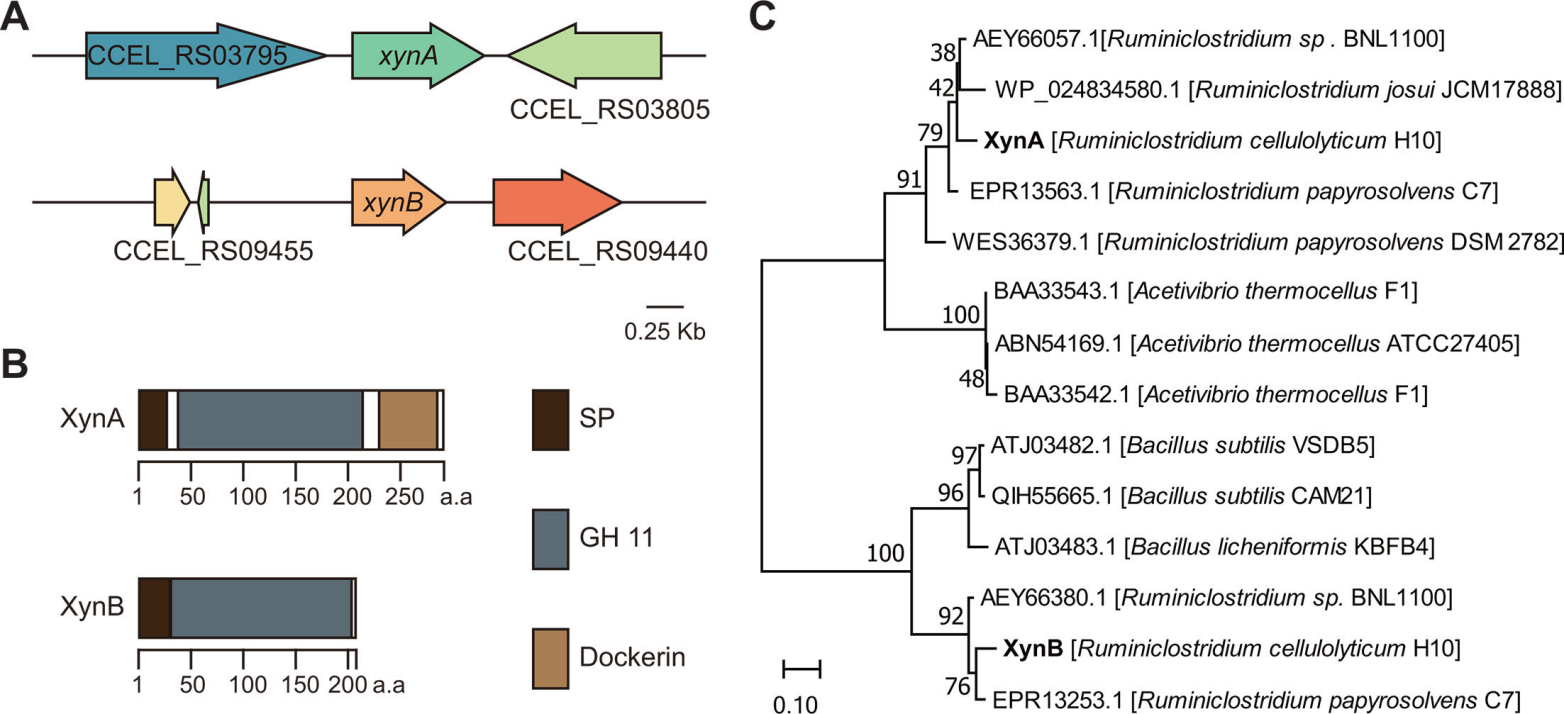
GH11 xylanase-encoding genes in *R. cellulolyticum*. (**A**) Genetic organization of *xynA* and *xynB* genes encoding xylanases. (**B**) Modular structure of GH11 XynA and XynB xylanases. (**C**) Phylogenetic analysis of GH11 xylanases, with the tree built using a maximum composite likelihood method based on neighbor-joining algorithms, supported by 100 bootstrap replicates. The scale bar indicates 0.10 amino acid residue substitutions per position.

Two putative genes encoding GH11 xylanases, CCEL_RS03800 and CCEL_RS09445, were predicted at two separate single-gene loci in the genome of *R. cellulolyticum*, respectively, named *xynA* and *xynB* (Fig. 1A). Both XynA and XynB are extracellular proteins by the signal peptide prediction. A Type I dockerin domain was predicted at the C-terminal (amino acids 236–292) of XynA, indicating that it is a cellulosomal subunit. In contrast, XynB lacks a dockerin domain, suggesting that it functions as a free enzyme (Fig. 1B). The alignment of the amino acid sequences of both xylanases identified 42.24% sequence identity between both GH11 domains. Phylogenetic analysis of XynA and XynB alongside previously studied xylanases in other bacteria (23–26) revealed that XynA and XynB fall into two distinct groups (Fig. 1C). XynA was identified in bacteria that are homologous to *R. cellulolyticum* and showed a close relationship with xylanases from anaerobic *Ruminiclostridium* species known to produce cellulosomes, suggesting that XynA is the orthologous enzyme within this bacterial group. In contrast, XynB was not found in *Ruminiclostridium papyrosolvens* DSM2782 and *Ruminiclostridium josui JCM17888*. Instead, it was found to group with enzymes from *Bacillus subtilis* and *Bacillus licheniformis*, which implies that XynB might be a paralog that was acquired via horizontal gene transfer.

### XynB is crucial for the utilization of xylan by *R. cellulolyticum*

**Fig 2:**
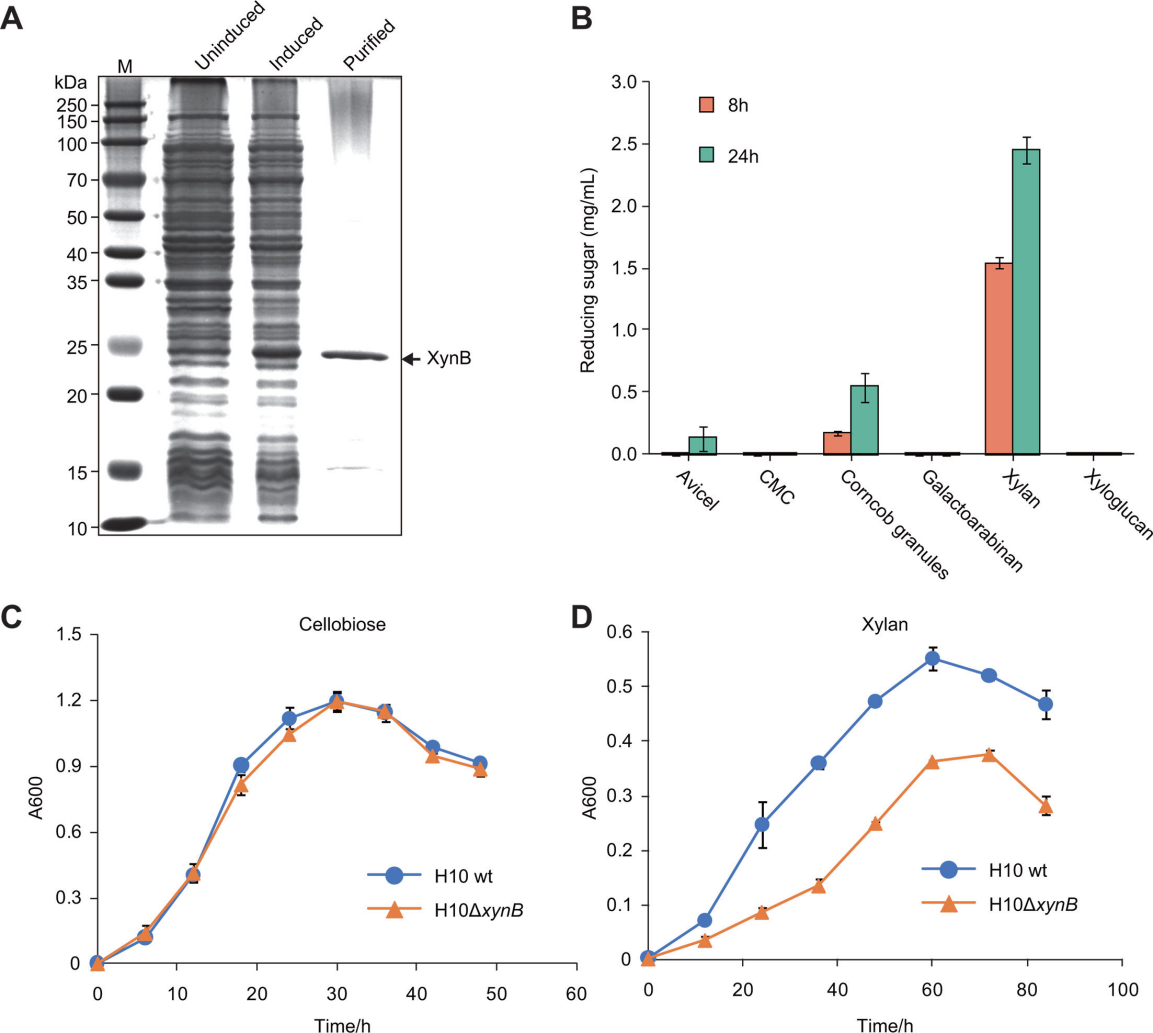
Functional characterization of XynB *in vitro* and *in vivo*. (**A**) SDS-PAGE analysis of the expression and purification of XynB. (**B**) Activity assay of recombinant XynB on various polysaccharides. (**C and D**) Growth curves of the *xynB* mutant on cellobiose (**C**) and xylan (**D**), compared to the wild type of *R. cellulolyticum*. All experiments were performed in triplicate and are shown with standard deviations.

To gain a better understanding of XynB, we further analyzed the function of XynB both *in vitro* and *in vivo*. Initially, we successfully achieved the heterologous expression and purification of recombinant XynB, although some leakage expression was observed (Fig. 2A). We then compared the enzymatic activities of XynB against an array of polysaccharides, including microcrystalline cellulose (Avicel), carboxymethyl cellulose, galactoarabinan, xyloglucan, xylan, and corncob granules. Our findings revealed that XynB exhibited activity specifically on xylan and corncob granules, confirming its classification as a xylanase. However, we also detected minimal exoglucanase activity on Avicel following 24 hour incubation. Notably, the amount of sugar released from corncob granules was significantly lower than that from xylan (Fig. 2B). This can be attributed to the fact that the xylan present in corncob is shielded by other components, which limit the accessibility of xylanases, despite the high xylan content in corncob (27).

Furthermore, the gene *xynB* was successfully disrupted by employing the xylan-inducible ClosTron (28). We screened for mutants (named H10Δ*xynB*) using Southern blot to investigate the off-target integrations. The results showed that the mutants had a single intron insertion, suggesting the target gene was disrupted without off-target effects. We then compared the growth of the H10Δ*xynB* mutant and wild-type strain on cellobiose and xylan by determining the cell turbidity of the culture. Both the wild type and the mutant showed similar growth patterns on cellobiose (Fig. 2C), whereas a slowdown of growth was observed for the *xynB*-disrupted strain when xylan was used as the carbon source (Fig. 2D). These findings demonstrate that XynB plays a pivotal role in the degradation of xylan by *R. cellulolyticum*.

### Transcription of *xynB* is induced by xylan and inhibited by cellobiose

**Fig 3:**
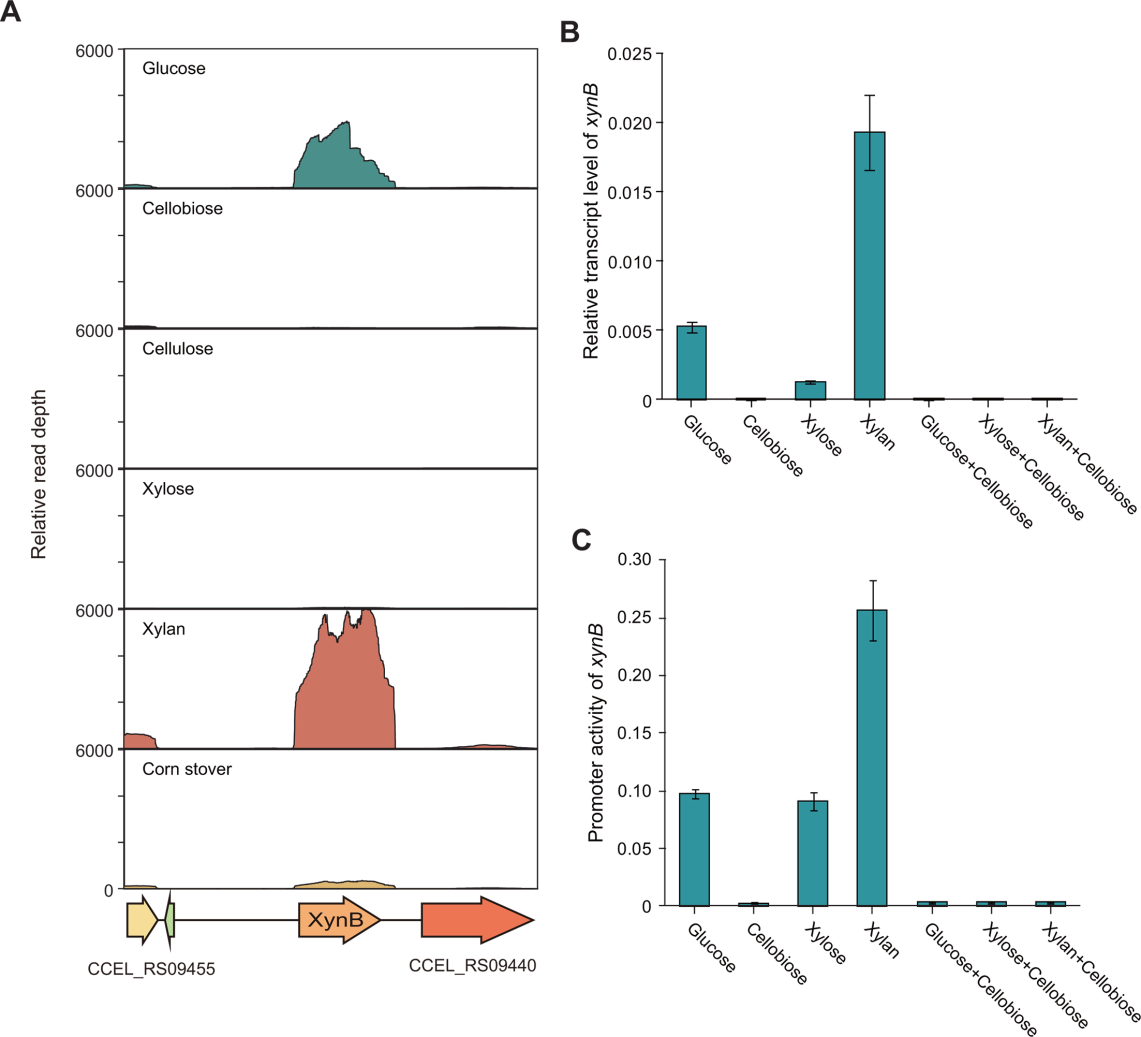
Transcriptional analysis of *xynB* under various carbon sources. (**A**) Comparison of transcription profiles of *xynB* under different carbon sources. (**B**) RT-qPCR analysis of *xynB* transcription in response to glucose, cellobiose, xylose, xylan, and their combinations with cellobiose. (**C**) Analysis of promoter activity of *xynB* under glucose, cellobiose, xylose, xylan, and their mixtures with cellobiose. All experiments were performed in triplicate and are shown with standard deviations.

The expression and regulation of *xynB* were further examined in *R. cellulolyticum*. Our previous transcriptomic data (29) showed that *xynB* was actively transcribed in the presence of xylan, glucose, or corn stover, but its transcription is significantly lower under cellobiose, cellulose, and xylose. Notably, the transcription level of *xynB* under xylan was higher than that of glucose, yet both xylan and glucose induced higher transcription levels of *xynB* than corn stover did (Fig. 3A).

Furthermore, the transcription level of *xynB* was determined by quantitative reverse transcription-PCR (qRT-PCR) under different carbon sources, including glucose, cellobiose, xylose, and xylan. The results of qRT-PCR confirmed that xylan induced the highest level of *xynB* transcription among the tested carbon sources. Specifically, the transcription level under glucose and xylose was significantly lower, at only 28% and 7%, respectively, of that observed under xylan. Notably, the transcription of *xynB* was virtually undetectable under cellobiose. These findings are consistent with the transcriptomic data, demonstrating that the transcription of *xynB* is induced by xylan, whereas it is repressed by cellobiose (Fig. 3B). However, when the carbon sources that induce the transcription of *xynB*, such as xylan, glucose, and xylose, were co-cultured with cellobiose, the transcription of *xynB* was found to be fully repressed. This finding suggests that cellobiose serves as a catabolite repressor, effectively inhibiting the expression of *xynB* (Fig. 3B).

Moreover, we evaluated the promoter activity of *xynB* in *R. cellulolyticum* using the GusA reporter system. A 962-nucleotide intergenic region was identified upstream from the *xynB* start codon and genetically fused to *gusA*. Strains of *R. cellulolyticum* carrying the *xynB* promoter-*gusA* fusion construct were, respectively, cultivated in glucose, xylose, xylan, and their mixtures with cellobiose and compared to cultivations with cellobiose alone. The results of the GusA assay showed complete agreement with the qRT-PCR results (Fig. 3C). Altogether, these findings demonstrate that the transcription of *xynB* is subject to dual regulation, being induced by xylan and repressed by cellobiose.

### The *xynB* gene is transcribed into two distinct transcripts

**Fig 4:**
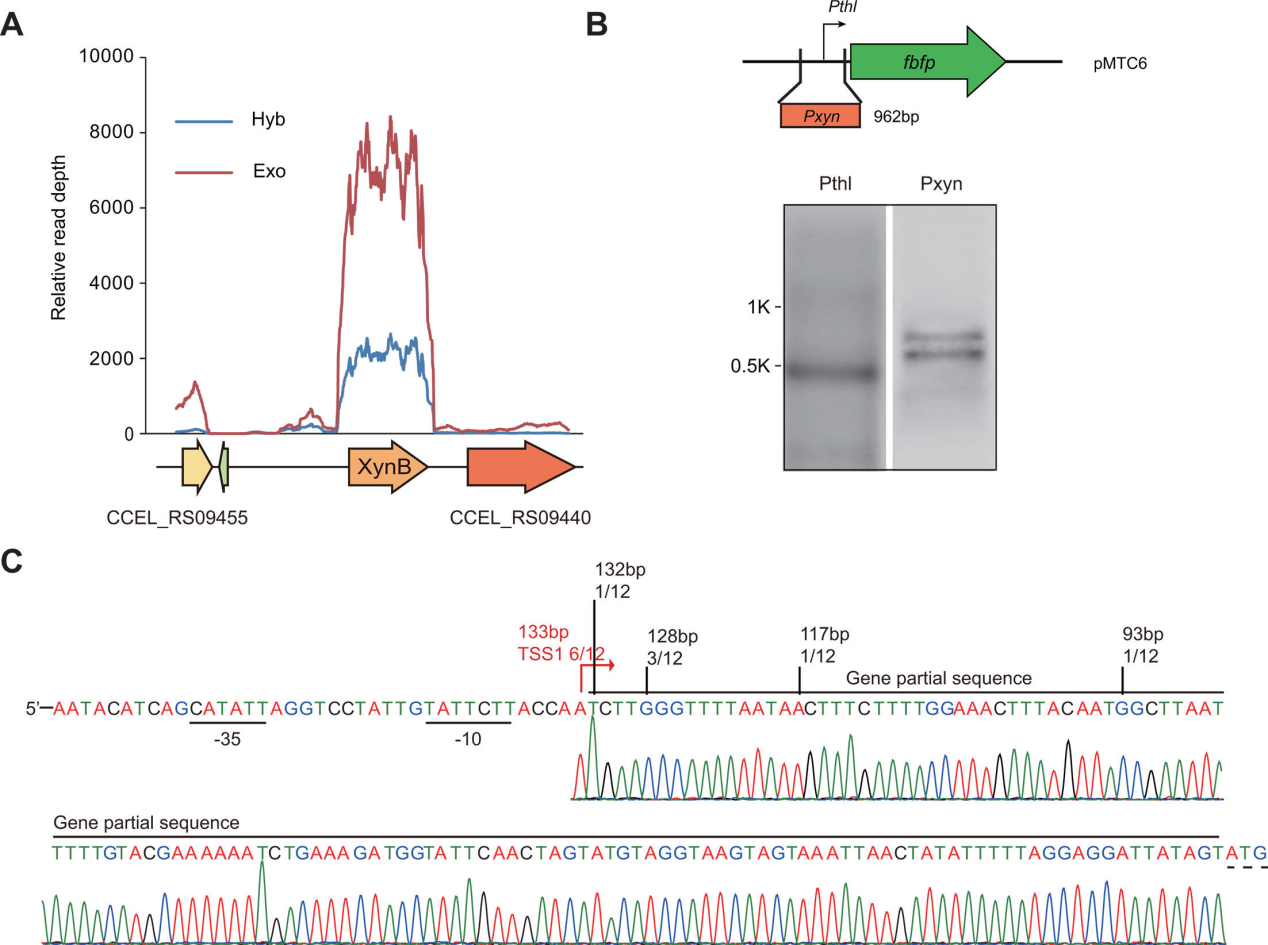
Analysis of the structure of the *xynB* transcripts. (**A**) Comparison of transcription landscapes of *xynB* from Hyb and Exo libraries. (**B**) Northern blot analysis of transcripts driven by the promoter region upstream of *xynB* (Pxyn). (**C**) Determination of TSS of *xynB* using 5′RACE.

The transcription profile of *xynB* from our previous differential RNA-sequencing (dRNA-seq) data (30) showed that the presence of a low-abundance transcript located in the 5′UTR of the *xynB* mRNA. This suggests that *xynB* is transcribed into two distinct types of transcripts: a short one and a long one. The short transcript could either be a processed form derived from the long primary transcript or be transcribed independently (Fig. 4A). We further fused the 962-nucleotide intergenic region upstream of *xynB* with the reporter gene *fbfp,* encoding an oxygen-independent green fluorescent protein, to investigate the transcriptional activity at the 5′UTR of *xynB*. Northern blot analysis indicated that two distinct bands were detected by the *fbfp* probe, confirming that the promoter region of *xynB* is capable of producing two different transcripts (Fig. 4B). However, when we introduced terminator exonuclease (denoted Exo) to digest the processed transcripts with 5′P but not 5′PPP primary transcripts, we found that the short transcript of *xynB* was not digested, suggesting the short transcript of *xynB* is also a primary transcript (Fig. 4A). Finally, we employed 5′-RACE to pinpoint the transcription start sites (TSSs) of the *xynB* transcripts. The analysis revealed that the TSS for the short transcript is located 133 bp upstream from the start codon of XynB. However, the TSS for the long transcript could not be determined due to the low abundance of this transcript (Fig. 4C).

**Fig 5:**
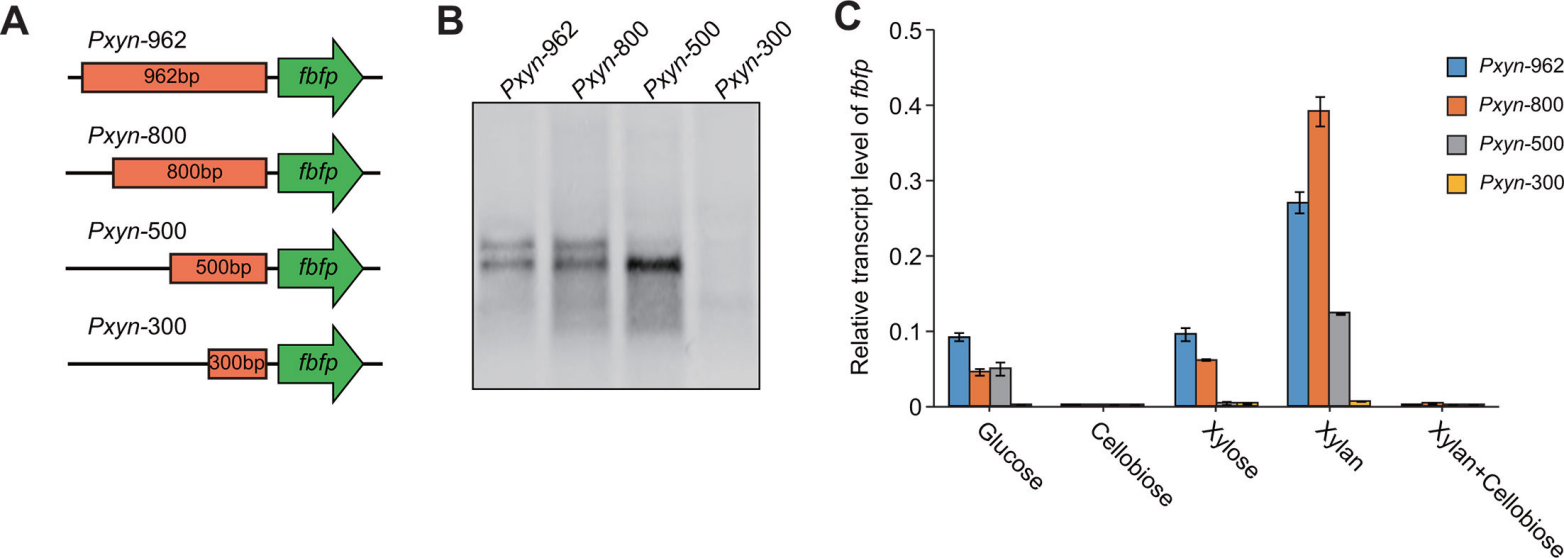
Deletion analysis of the *xynB* promoter region. (**A**) Construction of various transcriptional fusions by integrating different subregions of the *xynB* promoter upstream of *fbfp*. (**B**) Northern blot analysis of transcripts of *fbfp* driven by the different promoter regions upstream of *xynB*. (**C**) RT-qPCR analysis of *fbfp* transcription driven by the various promoter regions upstream of *xynB* under different carbon sources. All experiments were performed in triplicate and are shown with standard deviations.

We truncated the 962-nucleotide sequence to identify the sequences involved in regulating *xynB* promoter activity. The shortened sequences (800, 500, and 300 nucleotides in length) were also used to generate transcriptional fusions with the *fbfp* reporter gene (Fig. 5A). The transcription of *fbfp* was analyzed for bacteria cultured on xylan by Northern blot. It was revealed that Pxyn-800 also supported the production of two transcripts similar to the full-length Pxyn-962. In contrast, Pxyn-500 only promoted the transcription of the short transcript, and Pxyn-300 had lost the ability to initiate transcription (Fig. 5B). It was suggested that the region ranging from 300 to 500 bp plays a role in activating the promoter for the small transcript, while the region between 500 and 800 bp is involved in activating the promoter for the long transcript. Furthermore, the transcript level of *fbfp* was determined for bacteria cultured on cellobiose and/or xylan by qRT-PCR. The qRT-PCR results under xylan were consistent with the Northern blot findings, in which the transcript level of *fbfp* promoted by Pxyn-800 was equal to that of Pxyn-962, whereas the transcript level driven by Pxyn-500 was only half that of Pxyn-962, and *fbfp* was hardly transcribed under the control of Pxyn-300. The reduction in transcript level under Pxyn-500 may be caused by the inactivation of the promoter for the long transcript. Additionally, the transcription of *fbfp* was virtually undetectable in cultures on cellobiose or a mixture of cellobiose and xylan for all promoter regions, suggesting that the truncated Pxyn-800 and Pxyn-500 are still regulated by cellobiose-mediated catabolite repression (Fig. 5C).

### Transcription of *xynB* is activated by the xylan-sensing TCS

Our previous study indicated that carbohydrate-active enzymes (CAZymes) in *R. cellulolyticum*, which are specifically induced by substrates, are typically regulated by adjacent TCSs (29). We identified a specific TCS (CCEL_RS09435-09430) located downstream of *xynB* that could regulate the expression of *xynB*. To determine the TCS’s role in *xynB* regulation, we employed the ClosTron to disrupt the gene encoding the histidine kinase component of the TCS. Subsequently, we analyzed the transcription of *xynB* in the resulting mutant H10ΔRS09435. It was shown that in the H10ΔRS09435 mutant, *xynB* was repressed in the presence of cellobiose and induced by xylan, mirroring the behavior observed in the H10 wild-type strain (Fig. S1). These results suggest that the TCS located in proximity to *xynB* does not contribute to its transcriptional regulation.

**Fig 6:**
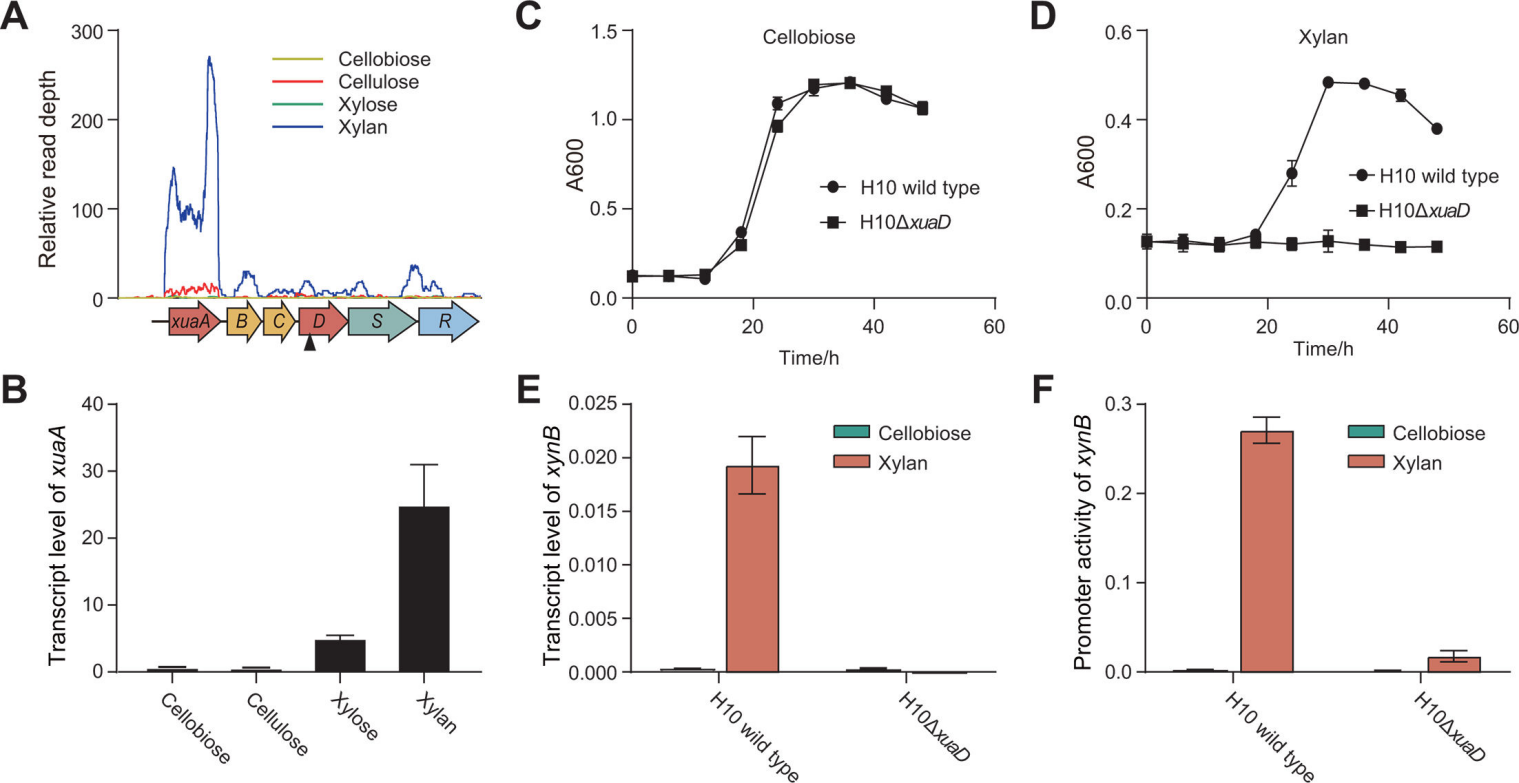
Induction of *xynB* transcription by TCS XuaDSR sensing xylan. (**A**) Comparison of transcription profiles of the *xua* gene cluster, encoding a TCS (*xuaDSR*) and an ABC transporter (*xuaABC*), under different carbon sources. (**B**) RT-qPCR analysis of transcription of *xuaA* encoding a sugar-binding protein of the ABC transporter. (**C and D**) Growth curves of the *xuaD* mutant on cellobiose (**C**) and xylan (**D**), compared to the wild type of *R. cellulolyticum*. (**E and F**) RT-qPCR analysis of *xynB* transcription (**E**) and promoter activity of *xynB* (**F**) in the *xuaD* mutant response to cellobiose and xylan, compared to the wild type of *R. cellulolyticum*. All experiments were performed in triplicate and are shown with standard deviations.

Our previous transcriptomic analysis identified a gene cluster encoding xylan-utilization-associated ABC transporter (CCEL_RS05685-05695, named *xuaABC*). This cluster was specifically induced by xylan and was potentially under the control of its downstream TCS (CCEL_RS05700-05710, named *xuaDRS*) (Fig. 6A). Quantitative PCR results confirmed that *xuaA,* encoding a sugar-binding protein, was selectively transcribed in the presence of xylan, aligning with the transcription profile of the *xuaABC* operon (Fig. 6B). To further investigate the role of XuaDRS, we constructed a mutant strain H10Δ*xuaD* by disrupting the *xuaD* gene (Fig. S2). Despite exhibiting an identical growth curve on cellobiose as the wild type (Fig. 6C), the H10Δ*xuaD* mutant was unable to grow on xylan, in contrast to the wild type, which could grow on xylan (Fig. 6D). This finding reveals the importance of the XuaDRS TCS in the signal sensing of xylan by *R. cellulolyticum*.

Interestingly, the transcription of *xynB* was hardly detectable under both cellobiose and xylan in the H10Δ*xuaD* mutant, indicating that the disruption of this TCS significantly impaired the activation of *xynB* transcription under xylan (Fig. 6E). To further validate the function of TCS XuaDRS, we compared the promoter activities of *xynB* in the H10Δ*xuaD* mutant by quantifying the transcription level of the reporter gene *fbfp*. Similarly, the promoter activities of *xynB* in H10Δ*xuaD* were significantly reduced under xylan, matching 6.6% of the levels observed in H10 wild type (Fig. 6F). Therefore, the TCS of XuaDRS is essential for sensing extracellular xylan signals and activating *xynB* transcription.

### Transcription of *xynB* is repressed by the cellobiose-sensing TCS

**Fig 7:**
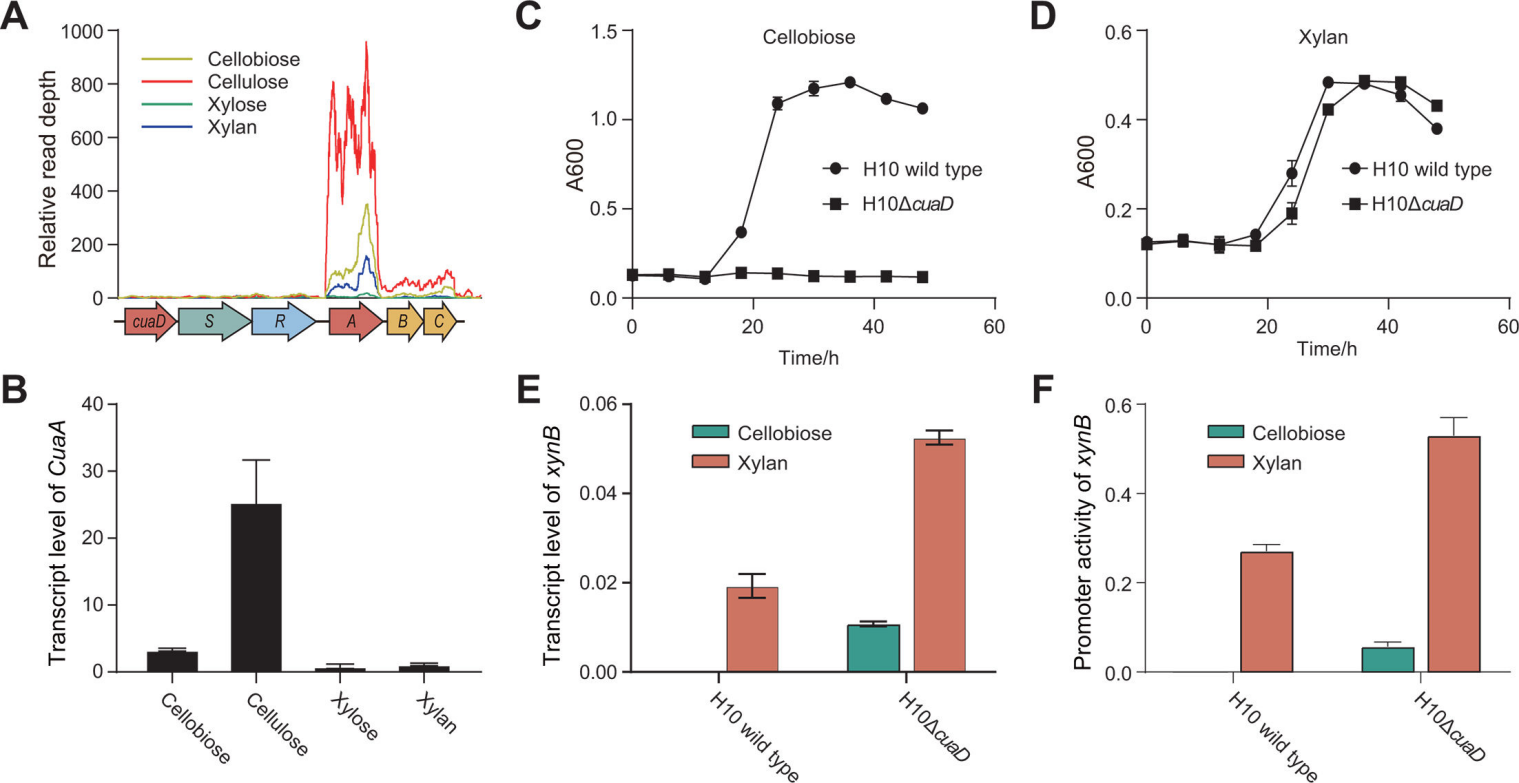
Repression of *xynB* transcription by TCS CuaDSR sensing cellobiose. (**A**) Analysis of transcription profiles of the *cua* gene cluster, which includes a TCS (*cuaDSR*) and an ABC transporter (*cuaABC*), under various carbon sources. (**B**) RT-qPCR analysis of transcription of *cuaA* encoding a sugar-binding protein of the ABC transporter. (**C and D**) Comparison of growth curves of the *cuaD* mutant on cellobiose (**C**) and xylan (**D**) with those of the wild type of *R. cellulolyticum*. (**E and F**) RT-qPCR analysis of *xynB* transcription (**E**) and promoter activity of *xynB* (**F**) in the *cuaD* mutant response to cellobiose and xylan, compared to the wild type of *R. cellulolyticum*. All experiments were performed in triplicate and are shown with standard deviations.

The expression of *xynB* in *R. cellulolyticum* is repressed by the presence of cellobiose. Our research has identified an additional gene cluster (*cuaDSR*) encoding a TCS that senses cellobiose to regulate the expression of downstream genes (*cuaABC*) responsible for the ABC transporter in *R. cellulolyticum*. The transcriptional analysis of this gene cluster revealed a specific upregulation in response to cellulose and cellobiose (Fig. 7A). Furthermore, quantitative PCR results substantiated that the *cuaA* gene, which encodes a sugar-binding protein, was preferentially transcribed in the presence of cellulose and cellobiose (Fig. 7B). This selective transcription pattern correlates with the transcriptional profile of the *cuaABC* operon. Consequently, we proceeded to investigate the potential regulatory role of the TCS in controlling the expression of *xynB*. To delve deeper into the function of the TCS CuaDRS, we generated a mutant strain, H10Δ*cuaD*, by disrupting the *cuaD* gene (Fig. S2). The growth curve analysis indicated that the disruption of *cuaD* led to an inability to grow on cellobiose (Fig. 7C), while growth on xylan remained unaffected (Fig. 7D). This observation underscores the pivotal role of the CuaDRS TCS in the sensing of cellobiose signals by *R. cellulolyticum*.

Curiously, under cellobiose conditions, the transcription of *xynB* in the H10Δ*cuaD* mutant was notably enhanced to exceed half the level seen in the H10 wild-type strain under xylan, whereas the transcription of *xynB* in the H10 wild type was hardly detected. Concurrently, under xylan conditions, the transcription of *xynB* in the H10Δ*cuaD* mutant was markedly elevated, nearly tripling that observed in the H10 wild type under the same conditions. These findings suggested that mutation of the TCS CuaDSR effectively eliminated the repressive effect of cellobiose on *xynB* transcription (Fig. 7E). To further confirm the function of TCS CuaDRS, we analyzed the promoter activities of *xynB* in the H10Δ*cuaD* mutant. Under xylan conditions, the promoter activities of *xynB* in H10Δ*cuaD* were significantly increased, reaching 200% of the levels observed in H10 wild type. Moreover, the promoter activities of *xynB* under cellobiose in H10Δ*cuaD* were detectable, whereas they were hardly detectable in the H10 wild type (Fig. 7F). Therefore, the TCS of CuaDRS is crucial for sensing extracellular cellulose-derived signals, such as cellobiose, and for repressing *xynB* transcription.

## DISCUSSION

The diversity of hemicelluloses found in plant biomass and the multitude of CAZymes required for their deconstruction has resulted in a less comprehensive understanding of bacterial hemicellulase-encoding gene regulation (31). Our previous transcriptomic analysis (29, 30) revealed that XynB, a free xylanase of the GH11 family in *R. cellulolyticum*, exhibits significantly higher expression levels compared to other CAZymes. This suggests an important role in the degradation of lignocellulosic biomass. Consequently, we investigated the mechanism of the expression and regulation of *xynB* in this study.

The activity assays of XynB confirmed its specificity for xylan, aligning with its role as xylanase. Our findings indicated that the expression of *xynB* is induced by both xylan and glucose. These results were not exactly consistent with those observed in other *Clostridium* species. For example, in *Clostridium termitidis*, the expression of genes encoding xylanases was found to be reliant on xylan, but not on xylose, cellobiose, or cellulose (32). In contrast, in *Clostridium phytofermentans*, the expression of xylanase-encoding genes was up-regulated when the organism was grown on both xylan and cellulose (33). This variation suggests that *Clostridium* species exhibited some divergence in their regulatory circuits for genes involved in xylanases.

**Fig 8:**
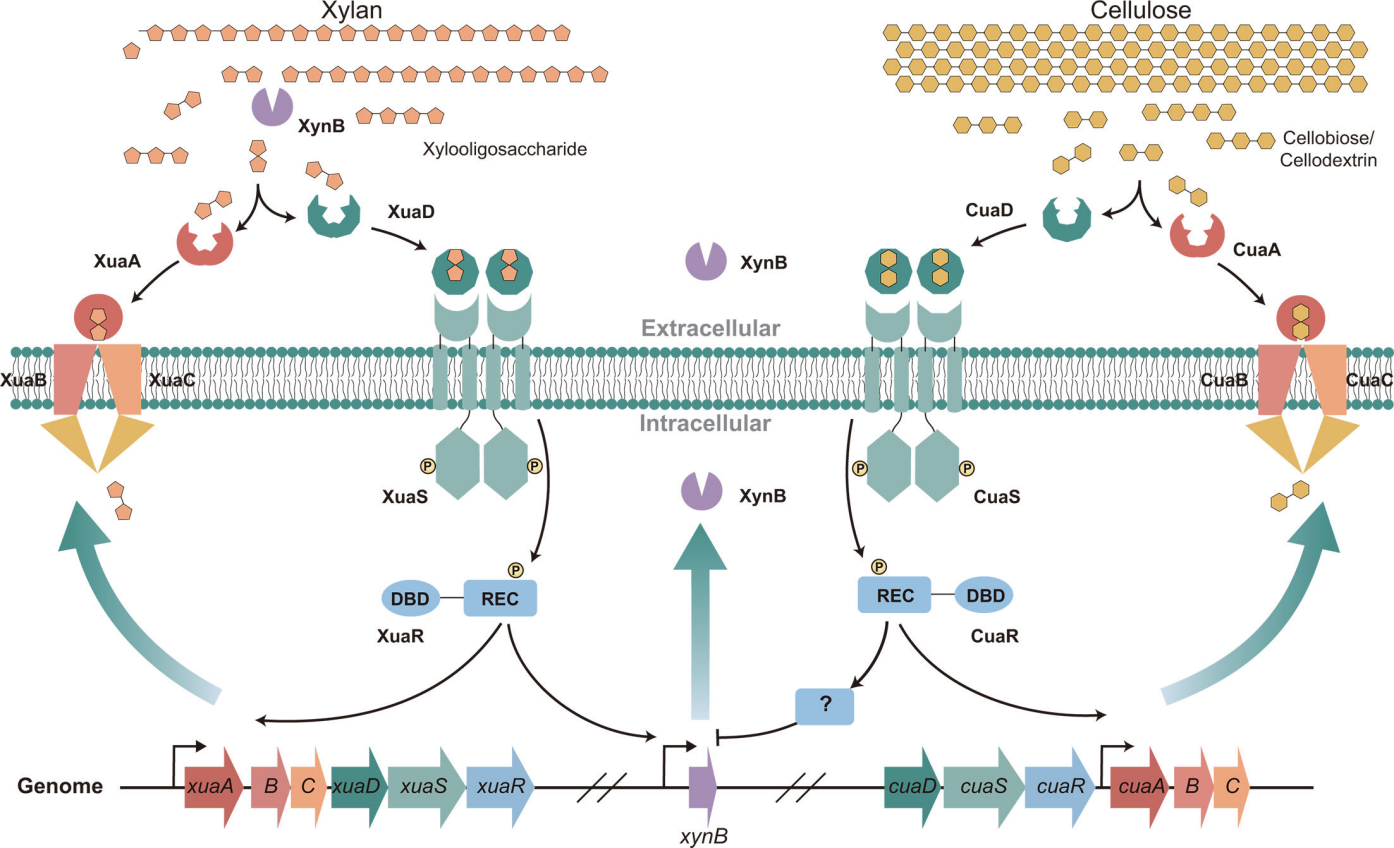
Cellular regulatory model of *xynB* transcription in *R. cellulolyticum*. The TCS XuaDSR detects extracellular xylan and triggers the activation of XynB and the ABC transporter XuaABC. XynB efficiently degrades xylan, and the resulting sugars are transported into the cell by XuaABC. Conversely, the TCS CuaDSR senses extracellular cellobiose, leading to the repression of XynB expression.

Previous studies on TCSs characterized the regulation of genes encoding xylanases, glucanases, arabinanases, and esterases across a spectrum of bacterial species, such as *Cellvibrio japonicus* (34) and *Bacteroides thetaiotaomicron* (35). In our previous research, we identified some CAZyme genes with substrate-specific expression patterns that were regulated by adjacent TCSs in *R. cellulolyticum* (29). For example, the *xyl-doc* gene cluster encoding hemicellulases was found to be under the control of the upstream TCS XygS/XygR (36). However, in the case of *xynB*, induction does not occur through nearby TCSs, such as CCEL_RS09434-09430, but is mediated by a distant TCS XuaDSR encoded by CCEL_RS05700-05710. These findings highlight the significant role that a single TCS can play in regulating the transcription of CAZymes, whether they are located in close proximity or at a genomic distance. It is intriguing to note that despite both the gene cluster *xuaABC* of ABC transporters downstream of *xuaDSR* and *xynB* being co-regulated by this TCS, we have been unable to identify conserved binding sites for the TCS in the promoters of these genes. This observation challenges our understanding of TCS-mediated regulation and suggests that additional, yet unidentified, mechanisms may be at play in the transcriptional control of these genes (Fig. 8).

Intriguingly, we observed significant repression of *xynB* expression in *R. cellulolyticum* by cellobiose, indicating that *xynB* is under the control of carbon catabolite repression (CCR). This contrasts with canonical CCR systems in model organisms like *Escherichia coli* and *B. subtilis*, where glucose serves as the primary catabolite repressor. In *R. cellulolyticum*, however, cellobiose appears to function as the preferred catabolite repressor, while glucose paradoxically induces *xynB* expression. This unique regulatory mechanism aligns with *R. cellulolyticum*’s ecological niche as a dedicated cellulose degrader. Our previous growth studies show this bacterium preferentially utilizes cellobiose and cellodextrin over glucose (29, 30). These cellulose-derived oligosaccharides serve as more efficient carbon sources since their β-1,4-glycosidic bond cleavage provides immediate energy, and they bypass the need for additional enzymatic processing to yield glucose monomers. Furthermore, the unexpected induction by glucose may reflect *R. cellulolyticum*’s need to sense and respond to complex plant biomass. As hemicellulose contains glucose in structural polymers like xyloglucans and glucomannans (4), glucose could serve as a biochemical indicator of hemicellulose availability, triggering *xynB* expression to initiate hemicellulose decomposition when cellulose-derived carbon sources become limiting.

A classic example of CCR-based regulation for hemicellulase-encoding genes is observed in *B. subtilis*, which uses both repressor catabolite control protein (CcpA) and the repressor GmuR (37). In the absence of glucose, the presence of cellobiose or mannobiose leads to the de-repression of mannanase-encoding genes due to limited phosphorylated HPr, which inactivates CcpA. GmuR, which requires phosphorylation by GmuA of the PTS, further controls these genes. The presence of glucomannan oligosaccharides results in unphosphorylated GmuA, leading to GmuR’s inability to repress transcription of the mannanase-encoding genes. Additionally, the co-regulation of genes encoding arabinanase and xylanase is observed, with two characterized repressors from the LacI family, AraR and XylR, functioning in tandem with canonical CCR (38). Surprisingly, the CCR of *xynB* in *R. cellulolyticum* is influenced by a TCS. We found that disrupting another TCS, encoded by *cuaDSR* (39), removes the inhibitory effect of cellobiose on *xynB* expression. This finding deviates from the established CCR mechanism involving CcpA. However, CuaDSR is proven to sense extracellular cellobiose and activate the cellobiose ABC transporter as transcription activators. Consequently, we speculate that this TCS may activate an unknown repressor, which, in turn, suppresses the expression of *xynB* (Fig. 8).

In conclusion, the expression and regulation of *xynB* in *R. cellulolyticum* is synergistically controlled by two TCSs: one that senses xylan and induces *xynB* expression, and another that senses cellobiose and represses *xynB* expression (Fig. 8). This regulatory mechanism may be attributed to the specific physiological properties of *R. cellulolyticum*, a cellulolytic bacterium that prefers cellobiose over glucose and employs ABC transporters to import extracellular sugars, rather than PTSs. The novel synergistic regulation highlights that *R. cellulolyticum* has evolved a complex metabolic strategy to enhance the efficiency of lignocellulosic saccharification in response to environmental changes.

## MATERIALS AND METHODS

### Bacterial strains and growth media

The bacterial strains and plasmids used in this experiment are listed in Table S1. *E. coli* DH5α served as the host strain for the construction of the recombinant plasmids and was grown at 37°C in Luria-Bertani broth (40). *R. cellulolyticum* ATCC35319 (H10) was cultured anaerobically at 35°C in GS-2 medium supplemented with 3 g/L of different sugars as the sole carbon source (41). When required, antibiotics were added at the following concentration: 20 µg/mL erythromycin or 100 µg/mL ampicillin. The solid medium was supplemented with 1.5% Agar.

### Generation of plasmids and recombinant strains

All plasmids constructed in this study are listed in Table S1. The isolation and manipulation of recombinant DNA were performed using standard techniques.

To predict the signal peptides of XynB, we utilized the Signal Peptide prediction tool available on the website (https://services.healthtech.dtu.dk/services/SignalP-5.0/) (42). The signal peptide was subsequently excluded for the primer design. The *R. cellulolyticum* H10 genome was used as the template for amplification of *xynB*. The target fragment was amplified using TransTaq-T DNA polymerase (TransGen, China) with primer pair pET28a-XynB-F/R. The resulting fragment was digested by BamHI and HindIII and ligated into the corresponding sites of pET28a, resulting in the construction of pET28a-XynB.

The shuttle vector pMTC6 between *E. coli* and *R. cellulolyticum* H10 contains an oxygen-independent FbFP expression cassette under the control of the thiolase (thl) promoter (Pthl) and terminator (43). We constructed plasmids with various truncated sequences of the *xynB* promoter to replace Pthl of pMTC6 using PstI and MluI for cloning. The target fragments for these constructs were amplified using primer pairs of P*xyn*-962-F/R, P*xyn*-800-F/R, P*xyn*-500-F/R, and P*xyn*-300-F/R, with the genome of *R. cellulolyticum* H10 serving as the template. Concurrently, these fragments were also designed to control the expression of β-glucuronidase GusA in pMTC14 derived from pMTC14 (44) for the analysis of promoter activity.

Plasmids designed for targeted gene disruption were constructed based on plasmid pSY6-Pxyl (28). Targeting sites for disruption and the intron-retargeting primers were designed using an online tool based on the Perutka algorithm (http://clostron.com/) (45). Gene SOEing (Splicing by Overlap Extension) method was performed to achieve the 353 bp targeting regions, with pSY6-Pxyl serving as the template (46). The plasmids pSY6-*xynB*/RS09435/*xuaD*/*cuaD* were constructed by inserting the corresponding targeted regions into the XhoI and BsrGI sites of pSY6-Pxyl. These plasmids were subsequently used for the disruption of *xynB*, Ccel_RS09435, *xuaD*, and *cuaD* genes in *R. cellulolyticum* H10.

### Electroporation of *R. cellulolyticum* H10

Electroporation was performed and optimized based on the previously described method (43). All manipulations were conducted under anaerobic conditions. Transformants carrying target plasmids were selected on the solid GS-2 medium supplemented with erythromycin.

### Mutant screening and plasmid curing

Monoclonal colonies were picked, and the insertion of the introns was verified to distinguish them from the wild type using PCR with primer pairs upstream and downstream of the target sites, because ClosTron inactivates the targeted gene by inserting a 900 bp intron at the target site. Finally, the mutant was inoculated in the medium without erythromycin pressure for plasmid curing.

### Quantitative reverse transcription-PCR

To determine the expression of *xynB* and *fbfp*, we employed qRT-PCR to measure the transcript abundance. Total RNA was isolated from *R. cellulolyticum* H10 cultures harvested at the mid-log phase using an EZ-10 Total RNA Mini-Prep Kit (Sangon, China). The cDNA was synthesized using the HiScript III RT SuperMix for qRT-PCR (+gDNA wiper) kit as described by the manufacturer’s instructions. The qRT-PCR reactions were performed with ChamQ Universal SYBR qPCR Master Mix (Vazyme, China) using the CFX96 real-time PCR detection system (Bio-Rad). The expression data were normalized relative to the transcript levels of 16sRNA. The primer sets used for qRT-PCR were listed in Table S1.

### Analysis of promoter activity

Cultures expressing GusA were chilled and centrifuged at 6,000 × *g* for 10 min at 4°C. Cell pellets were washed with three ice-cold Tris-EDTA buffer (10 mM Tris-HCl, 1 mM EDTA, pH 8.0) and resuspended in GusA buffer (50 mM sodium phosphate, 1 mM EDTA, pH 7.0) for ultrasonication. The total protein concentrations of enzyme samples were determined by the BCA Protein Assay Kit (Sangon, China).

GusA activity was determined using the fluorogenic substrate 4-methylumbelliferyl-β-D-glucuronic acid (MUG). Reaction mixtures containing diluted enzyme samples and MUG were incubated at 37°C and fluorescence was measured using a fluorescence spectrophotometer (PerkinElmer LS55, Waltham, USA). The kinetic curve was recorded at 365 nm excitation and 455 nm emission every 30 seconds for 5 min. GusA activity was calculated from the curve slope and protein concentration (U/mg).

### Southern hybridization

Southern blotting was performed to identify intron insertion in the genomic DNA of *R. cellulolyticum* H10 mutants, following the protocol detailed in previous literature (47). Genomic DNA was isolated and digested with HindIII at 37°C overnight. The resulting nucleic acids were transferred onto nylon membrane (Hybond-NX, GE Healthcare, USA) and hybridized with a Cy5.5-labeled probe specific to the intron sequence. This hybridization was performed in the hybridization buffer (ULTRAhyb, Thermo Fisher Scientific, USA) at 42°C overnight. The probe labeled by Cy5.5 at the 5′ end was prepared commercially (Sangon, China). The signals were detected with an Odyssey CLx dual-color infrared laser imaging system (LI-COR, USA) at 720 nm.

### Northern hybridization

Total RNA was isolated from *R. cellulolyticum* using an EZ-10 Total RNA Mini-Prep Kit (Sangon, China). The RNA quality was determined using a NanoVue Plus spectrophotometer (Biochrom, UK). Two micrograms of RNA samples were separated via electrophoresis on 1% denaturing agarose-formaldehyde gel and blotted onto a *N* + nylon membrane (Hybond-NX, GE HealthCare, USA). For the detection of Fbfp, Cy5.5-labeled DNA probes were prepared commercially (Sangon, China). The signals were captured using an Odyssey CLx dual-color infrared laser imaging system (LI-COR, USA) at 720 nm.

### 5′-RACE

According to the requirements of the 5′-RACE Kit (Sangon, China), 5′RACE-specific reverse transcription primers were designed. Total RNA of *R. cellulolyticum* H10 harboring the pXYN962 was extracted and amplified according to the kit’s standard protocol. The amplified fragment was cloned in T-Vector using the pMD19-T Vector Cloning Kit (TAKARA, China), and the white monoclonal colony was selected for PCR identification. The TSS was identified by sequencing.

## Data Availability

The data associated with this article were deposited in the National Center for Biotechnology Information Gene Expression Omnibus (GEO) under accession number GSE57652. The data are publicly accessible via the following link: https://www.ncbi.nlm.nih.gov/geo/query/acc.cgi?acc=GSE57652.

## SUPPLEMENTAL MATERIAL

Supplemental figures and tables aem.00062-25-s0001.docx Fig. S1 and S2 showing gene mutations and Tables S1 and S2 listing strains, plasmids, and primers used in this study.

ASM does not own the copyrights to Supplemental Material that may be linked to, or accessed through, an article. The authors have granted ASM a non-exclusive, world-wide license to publish the Supplemental Material files. Please contact the corresponding author directly for reuse.
