# Supplementary material for: Expression of xylanase XynB is synergistically controlled by two two-component systems in Ruminiclostridium cellulolyticum
Source: Appl Environ Microbiol. 2025 May 30;91(6):e00062-25. doi: 10.1128/aem.00062-25 (PMC12175519; doi:10.1128/aem.00062-25)
Supplement: Supplemental figures and tables — Fig. S1 and S2 showing gene mutations and Tables S1 and S2 listing strains, plasmids, and primers used in this study. [file aem.00062-25-s0001.docx]

***Expression of xylanase XynB is synergistically controlled by two two-component systems in Ruminiclostridium cellulolyticum***

Wenhao Zhang^1,2*^, Zili Qiu^3*^, Qiuyun Zhao, Ziyi Liu, Xiaorong Zhang^2^, Houhui Song^1§^, Chenggang Xu^1^^§^

^1^College of Animal Science and Technology & College of Veterinary Medicine of Zhejiang A&F University, Key Laboratory of Applied Technology on Green-Eco-Healthy Animal Husbandry of Zhejiang Province, Zhejiang Provincial Engineering Research Center for Animal Health Diagnostics & Advanced Technology, Zhejiang International Science and Technology Cooperation Base for Veterinary Medicine and Health Management, Hangzhou 311300, Zhejiang Province, China

^2^Key Laboratory of Chemical Biology and Molecular Engineering of Ministry of Education, Institute of Biotechnology, Shanxi University, Taiyuan 030006, Shanxi Province, China

^3^Institute of Applied Chemistry, Shanxi University, Taiyuan 030006, Shanxi Province, China

^*^These authors contributed equally to this work.

^§^To whom correspondence should be addressed. Tel. +86 571 63741392; Email: xucg@zafu.edu.cn

**SUPPLEMENTARY MATERIAL**

**TABLE S1** Bacterial strains and plasmids used in this study.


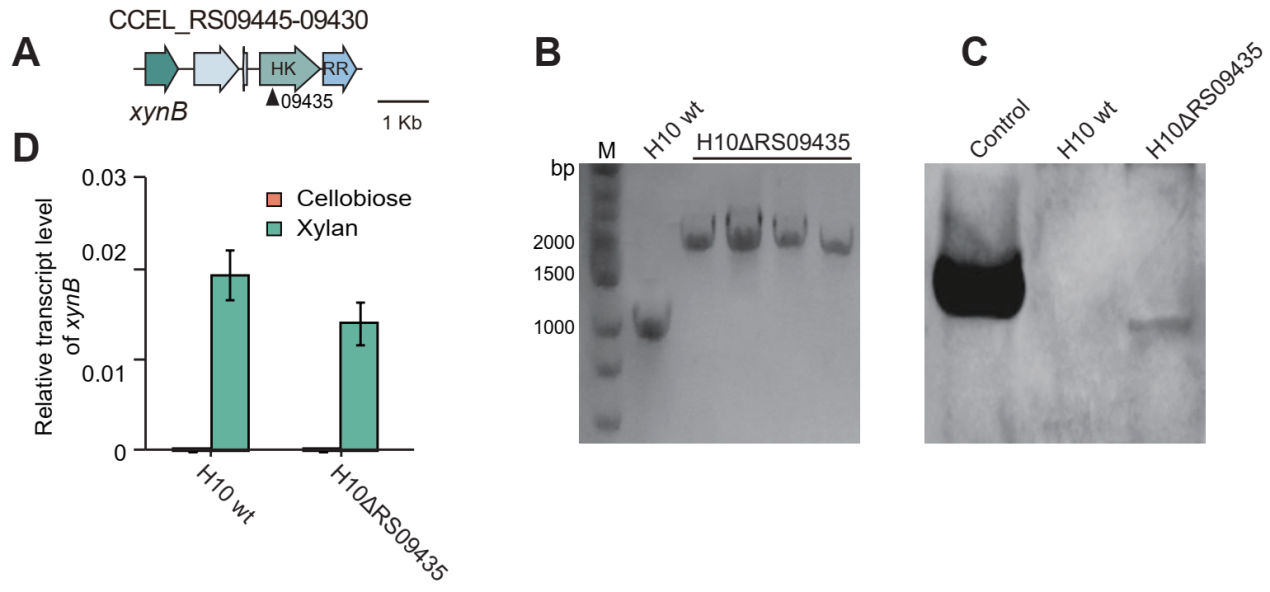


**Fig S1** Effect of TCS (CCEL_RS09435-09430) located downstream of *xynB* on the expression of *xynB*. (A) Genetic organization of TCS encoded by CCEL_RS09435-09430 and *xynB*. (B and C) Identification of TCS mutant (H10 H10ΔRS09435) by PCR (B) and Northern blot (C). (D) RT-qPCR analysis of *xynB* transcription in the mutant response to cellobiose and xylan, compared to the wild type of *R. cellulolyticum*.


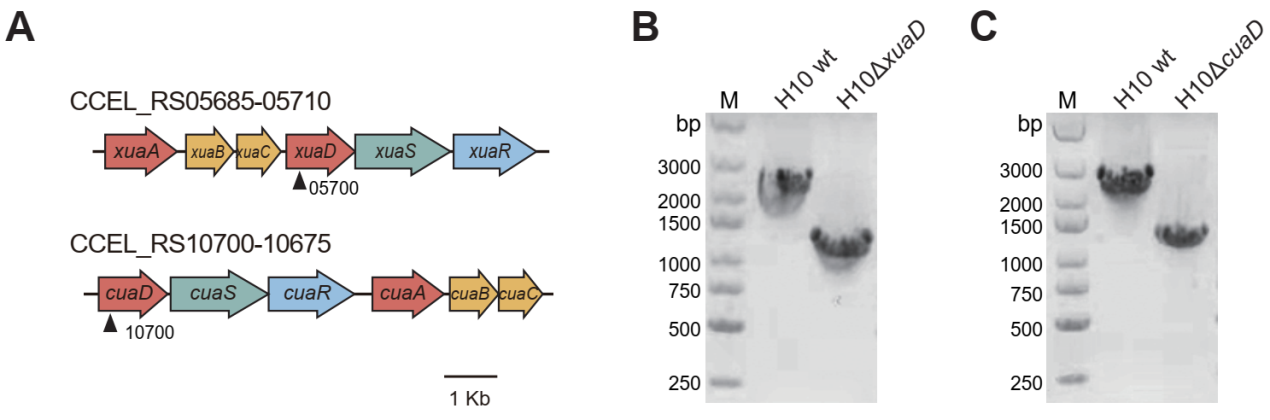


**Fig S2** Construction of TCS mutants H10Δ*xuaD* and H10Δ*cuaD*. (A) Genetic organization of the gene clusters harboring TCS XuaDSR and CuaDSR. Genes encoding encoding TCSs are flanked by genes ABC transporters. Closed triangles indicated the mutated genes. (B and C) PCR Identification of TCS mutants (H10Δ*xuaD* and H10Δ*cuaD*) disrupted by ClosTron.

**TABLE S1** Bacterial strains and plasmids used in this study

| Strain or plasmid | Genotype and/or relevant characteristics | Reference or source |
| --- | --- | --- |
| **Strain** |  |  |
| *R. cellulolyticum* H10 | Wild type | ATCC |
| *R. cellulolyticum* H10 Δ*mspI* | Derived from *R. cellulolytiucm* H10 with the *mspI* deleted | Granted from Cui Qiu et al. (1) |
| *R. cellulolyticum* H10 Δ*xynB* | *R. cellulolyticum* H10Δ*mspI* knockout gene *xynB* | This work |
| *R. cellulolyticum* H10 Δ*xuaD* | *R. cellulolyticum* H10Δ*mspI* knockout gene *xuaD* | This work |
| *R. cellulolyticum* H10 Δ*cuaD* | *R. cellulolyticum* H10Δ*mspI* knockout gene *cuaD* | This work |
| *E. coli* DH5α | Host cells for genes cloning and plasmids amplification | Transgene |
| *E. coli* BL21(plys) | Host cells for expressing protein | Transgene |
| **Plasmid** |  |  |
| pET28a | T7 ptomoter, His, Kan^R^ | Stored in our lab |
| pET28a-XynB | Expresse protein XynB in *E.coli* BL2 | This work |
| pMTC14 | Mls^R^, Amp^R^, *E. coli- R. cellulolytiucm* H10 shuttle vector bearing gusA gene, P4 promoter | This work |
| pMTC14-p*xyn* | pMTC14 derivative expression vector harboring p*xyn* | This work |
| pSY6 | Mls^R^, Amp^R^, *E. coli- R. cellulolytiucm* H10 shuttle vector, *ptb* promoter, containing *L. lactis* L1.LtrB intron and *ltrA* | Heap et al. (2) |
| pSY6-*xynB* | Derived from pSY6, targeting the *xynB* in *R. cellulolyticum* H10Δ*mspI* | This work |
| pSY6-RS09435 | Derived from pSY6, targeting the CCEL_RS09435 in *R. cellulolyticum* H10Δ*mspI* | This work |
| pSY6-*xuaD* | Derived from pSY6, targeting the *xuaD* in *R. cellulolyticum* H10Δ*mspI* | This work |
| pSY6-*cuaD* | Derived from pSY6, targeting the *cuaD* in *R. cellulolyticum* H10Δ*mspI* | This work |
| pMTC6 | Derived from pIMP1, MLS^R^, Amp^R^, *E. coli- R. cellulolytiucm* H10 shuttle vector, containing *PpFbFpm*, *lac* operator, *thl* promoter, *thl* terminator | Granted from Cui Qiu et al. (1) |
| pXYN962 | pMTC6 derivative expression vector harboring p*xyn*-962 | This work |
| pXYN800 | pMTC6 derivative expression vector harboring p*xyn*-800 | This work |
| pXYN500 | pMTC6 derivative expression vector harboring p*xyn*-500 | This work |
| pXYN300 | pMTC6 derivative expression vector harboring p*xyn*-300 | This work |

**REFERENCES**

1. Cui GZ, Hong W, Zhang J, Li WL, Feng Y, Liu YJ, Cui Q. 2012. Targeted gene engineering in Clostridium cellulolyticum H10 without methylation. J Microbiol Methods 89:201-8.

2. Heap JT, Pennington OJ, Cartman ST, Carter GP, Minton NP. 2007. The ClosTron: a universal gene knock-out system for the genus Clostridium. J Microbiol Methods 70:452-64.
